# Akbu-LAAO exhibits potent anti-tumor activity to HepG2 cells partially through produced H_2_O_2_ *via* TGF-β signal pathway

**DOI:** 10.1038/srep18215

**Published:** 2015-12-14

**Authors:** Chunmei Guo, Shuqing Liu, Panpan Dong, Dongting Zhao, Chengyi Wang, Zhiwei Tao, Ming-Zhong Sun

**Affiliations:** 1 Department of Biotechnology, Dalian Medical University, Dalian, Liaoning, 116044, China; 2 Department of Biochemistry, Dalian Medical University, Dalian, Liaoning, 116044, China

## Abstract

Previously, we characterized the biological properties of Akbu-LAAO, a novel L-amino acid oxidase from *Agkistrodon blomhoffii ussurensis* snake venom (SV). Current work investigated its *in vitro* anti-tumor activity and underlying mechanism on HepG2 cells. Akbu-LAAO inhibited HepG2 growth time and dose-dependently with an IC_50_ of ~38.82 μg/mL. It could induce the apoptosis of HepG2 cells. Akbu-LAAO exhibited cytotoxicity by inhibiting growth and inducing apoptosis of HepG2 as it showed no effect on its cell cycle. The inhibition of Akbu-LAAO to HepG2 growth partially relied on enzymatic-released H_2_O_2_ as catalase only partially antagonized this effect. cDNA microarray results indicated TGF-β signaling pathway was linked to the cytotoxicity of Akbu-LAAO on HepG2. TGF-β pathway related molecules *CYR61, p53, GDF15, TOB1, BTG2, BMP2, BMP6, SMAD9, JUN, JUNB, LOX, CCND1, CDK6, GADD45A, CDKN1A* were deregulated in HepG2 following Akbu-LAAO stimulation. The presence of catalase only slightly restored the mRNA changes induced by Akbu-LAAO for differentially expressed genes. Meanwhile, LDN-193189, a TGF-β pathway inhibitor reduced Akbu-LAAO cytotoxicity on HepG2. Collectively, we reported, for the first time, SV-LAAO showed anti-tumor cell activity *via* TGF-β pathway. It provides new insight of SV-LAAO exhibiting anti-tumor effect *via* a novel signaling pathway.

The L-amino acid oxidase (LAAO, EC 1.4.3.2) are flavoenzymes catalyzing the stereospecific oxidative deamination of L-amino acids to produce α-keto acids, ammonia and H_2_O_2_^1^,^2^,^3^. As one major snake venom (SV) component, LAAO commonly exists as homodimeric FAD-(flavin adenine dinucleotide) or FMN-(flavin mono-nucleotide) glycoprotein^4^,^5^,^6^. The anti-microbial, anti-platelet and anti-tumor functions^7^,^8^,^9^,^10^,^11^,^12^,^13^,^14^,^15^ of SV-LAAOs were commonly reported to be mediated by enzymatic- released H_2_O_2_^16^,^17^,^18^. However, the underlying action mechanisms are still unclear.

Previously, we purified a novel LAAO from *Agkistrodon blomhoffii ussurensis* snake venom, named as Akbu-LAAO. It is a homodimeric glycoprotein with a size of ~124.4 kDa with apparent anti-platelet aggregation and anti-bacterial activities^16^. In current study, we investigated the tumor suppression effect and underlying action mechanism of Akbu-LAAO to HepG2 cells. It inhibited the proliferation and induced the apoptosis of HepG2 cells, which was revealed only partially associated with the enzymatic-released H_2_O_2_. Interestingly, the results from cDNA microarray and qRT-PCR assays indicated Akbu-LAAO showing cytotoxicity to HepG2 cells *via* TGF-β signaling pathway that was for the first time linked to the action of SV-LAAOs on tumor cells.

## Results

### Akbu-LAAO inhibits *in vitro* growth of HepG2 cell

**Figure 1:**
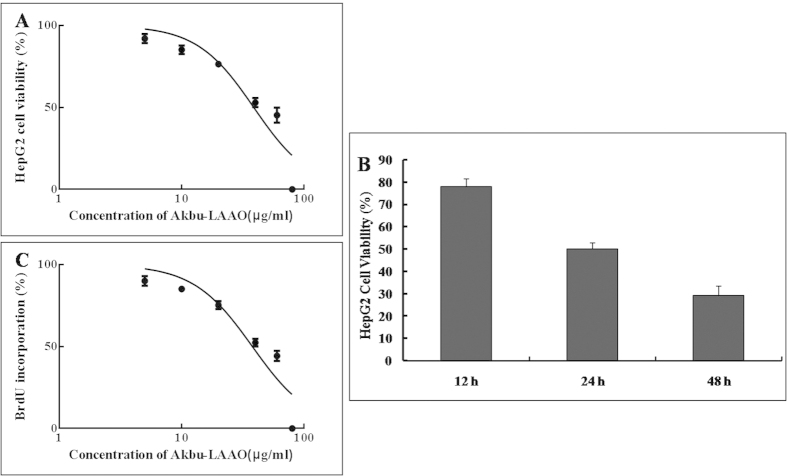
Akbu-LAAO inhibits the *in vitro* proliferation of HepG2. (**A**) MTT assay indicated Akbu-LAAO treatment for 24 h dose-dependently inhibited HepG2 proliferation. (**B**) The administration of 38.82 μg/mL Akbu-LAAO time-dependently inhibited HepG2 growth. (**C**) BrdU assay showed Akbu-LAAO treatment for 24 h dose-dependently inhibited HepG2 proliferation.

The effects of Akbu-LAAO on the viability and proliferation of HepG2 cells were determined using MTT and BrdU methods. Akbu-LAAO showed clear cytotoxicity on HepG2 by inhibiting cell viability in a dose- (Fig. 1A) and time- dependent (Fig. 1B) manner. An IC_50_ of ~38.82 μg/mL was measured for Akbu-LAAO on HepG2 viability in 24 h (Fig. 1A). Akbu-LAAO reduced *in vitro* proliferation of HepG2 dose-dependently (Fig. 1C). BrdU assay showed the BrdU incorporation during DNA synthesis in proliferating HepG2 cells was suppressed in the presence of Akbu-LAAO. With the administration for 24 h, an IC_50_ of ~37.49 μg/mL was measured for Akbu-LAAO on HepG2 proliferation. Akbu-LAAO administration dosage of 38.82 μg/mL was selected for following experiments.

### Catalase scavenging partially suppresses the cytotoxicity of Akbu-LAAO on HepG2 cell

**Figure 2:**
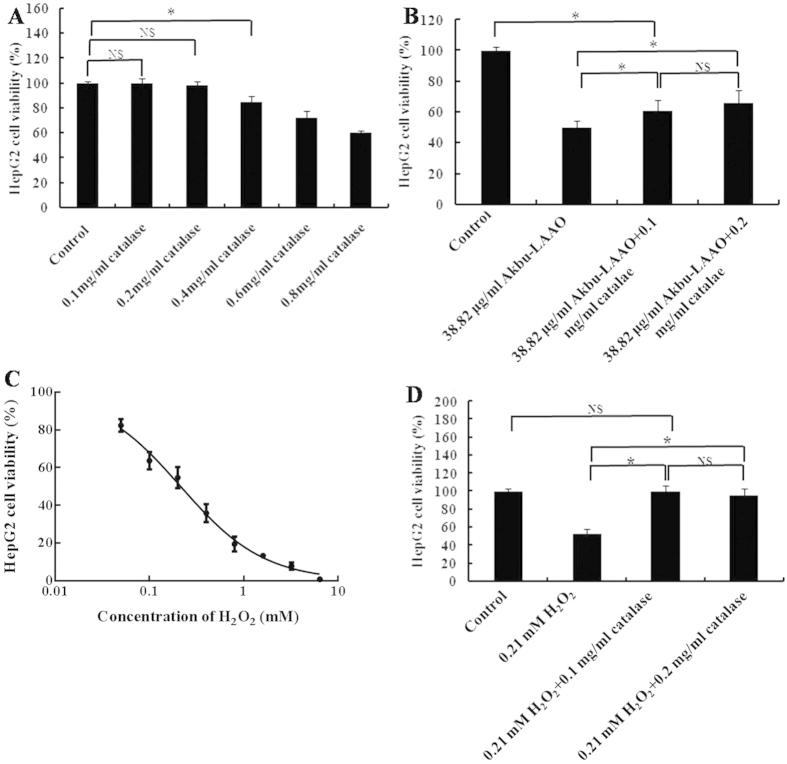
Catalase scavenging influences on the cytotoxicities of Akbu-LAAO and exogenous H_2_O_2_. (**A**) The effect of catalase on HepG2 proliferation. (**B**) The influence of catalase on Akbu-LAAO cytotoxicity to HepG2. (**C**) Exogenous H_2_O_2_ inhibited HepG2 proliferation. (**D**) The influence of catalase on exogenous H_2_O_2_ cytotoxicity to HepG2. All experiments were performed in triplicate, * denotes *P* < 0.05

Catalase is a scavenger of H_2_O_2_. At the concentration of 0.1 and 0.2 mg/mL, catalase showed no apparent toxicity to HepG2 cells, while, relative higher concentrations of catalase showed cytotoxicity (Fig. 2A). In current work, we selected 0.1 and 0.2 mg/mL catalase for further experiments. 0.2 mg/mL of catalase decreased the cytotoxicity of 24 h administration of 38.82 μg/mL Akbu-LAAO on HepG2 cells by ~30%. (Fig. 2B). The IC_50_ of exogenous H_2_O_2_ administration for 24 h on HepG2 was ~0.21 mM (Fig. 2C). 0.1 mg/mL of catalase treatment could completely abolish the cytotoxicity of H_2_O_2_ on HepG2 (Fig. 2D). The proliferation inhibition of Akbu-LAAO on HepG2 was not solely contributed by the enzymatic-released H_2_O_2_. It can be concluded the action of Akbu-LAAO on HepG2 proliferation differs from that of exogenous H_2_O_2_. H_2_O_2_ production is not fully responsible for the cytotoxicity of Akbu-LAAO on HepG2.

### Akbu-LAAO alters the cellular morphology of HepG2

**Figure 3:**
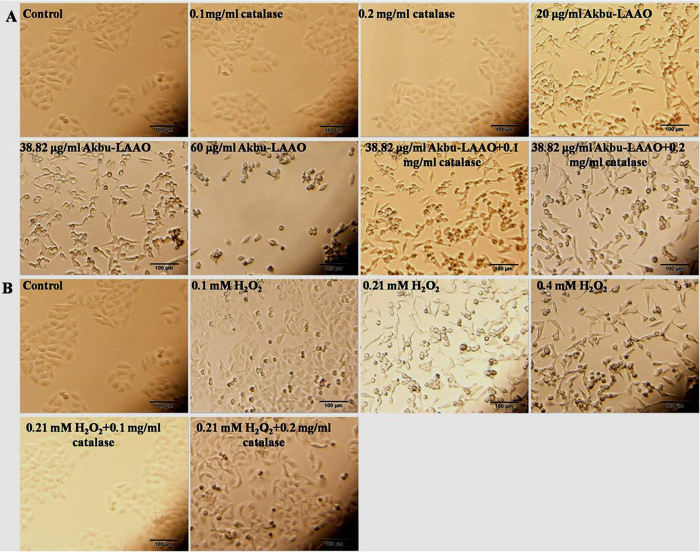
The influences of Akbu-LAAO and exogenous H_2_O_2_ administrations on HepG2 morphology. (**A**) HepG2 morphology observation following Akbu-LAAO administration in the presence and absence of catalase. (**B**) HepG2 morphology observation following exogenous H_2_O_2_ administration in the presence and absence of catalase. Cell images were taken using an inverted light microscope at the magnification of 100×.

The cell population decreased following the concentration increases of Akbu-LAAO (Fig. 3A) and exogenous H_2_O_2_ (Fig. 3B). 0.1 and 0.2 mg/mL of catalase showed no effect on HepG2 cell morphology. Both Akbu-LAAO and H_2_O_2_ treatments could does-dependently cause the cytoplasmic vacuolation, shrinkage, detachment from culture matrix and death of HepG2. Catalase could not restore the morphological change of HepG2 induced by Akbu-LAAO (Fig. 3A), while, it completely reversed the morphological changes of HepG2 induced by H_2_O_2_ (Fig. 3B). These results confirmed that the action of Akbu-LAAO on HepG2 cells differed from that of exogenous H_2_O_2_.

### Akbu-LAAO alters the ultrastructure of HepG

**Figure 4:**
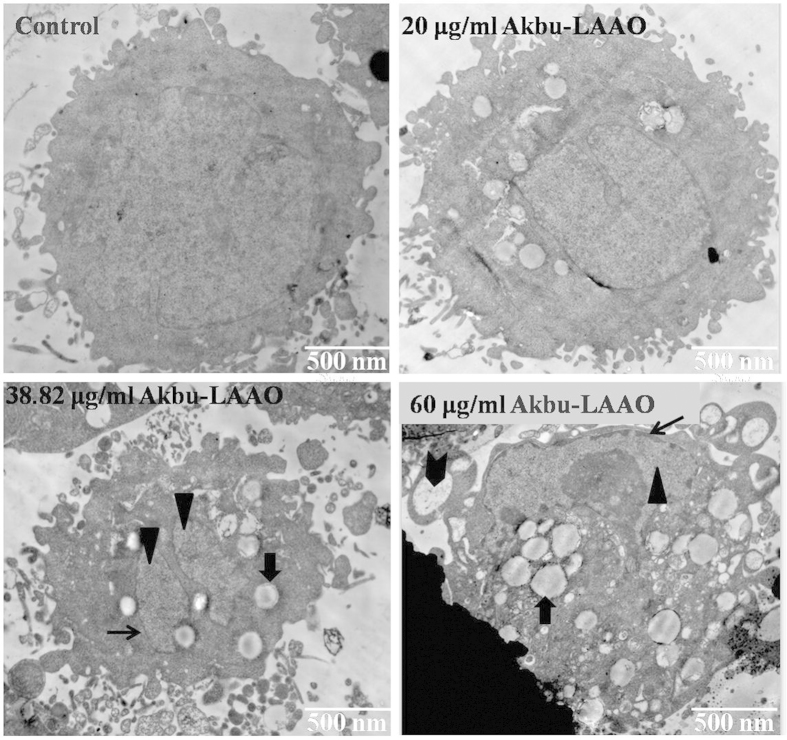
Transmission electron microscopy ultrastructural characterization of HepG2 to Akbu-LAAO stimulation. Images were obtained at a magnification of 12000×. 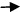 represents chromatin condensation, 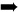 represents cytoplasmic vacuolation, 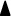 represents nucleolus structure disorganization, 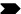 represents apoptotic bodies.

The ultrastructure alteration of HepG2 cells induced by Akbu-LAAO administration was tracked by TEM. Akbu-LAAO induced HepG2 apoptosis. As shown in Fig. 4, surface microvillis were diminished in HepG2 cells following Akbu-LAAO treatment. In the presence of 20 μg/mL of Akbu-LAAO, HepG2 showed no clear ultrastructural changes. Typical ultrastructural characteristics for early-stage apoptosis such as cytoplasmic vacuolation (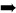, Fig. 4), nucleolus structural disorganization (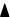, Fig. 4), and chromatin condensation (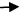, Fig. 4) at nuclei margins were clearly observed for HepG2 cells following Akbu-LAAO treatment with 38.82 μg/mL. 60 μg/mL treatment of Akbu-LAAO caused the ultrastructural changes of late-stage apoptosis, advanced structural disorganization, chromatin condensation in inner-nuclear margin, cell and nucleus disaggregation and increase of apoptotic bodies (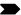, Fig. 4 ) for HepG2 cells. These indicated Akbu-LAAO could induce the ultrastructure alteration and apoptosis of HepG2 cells.

### Akbu-LAAO induces HepG2 apoptosis differs from exogenous H_2_O_2_

**Figure 5:**
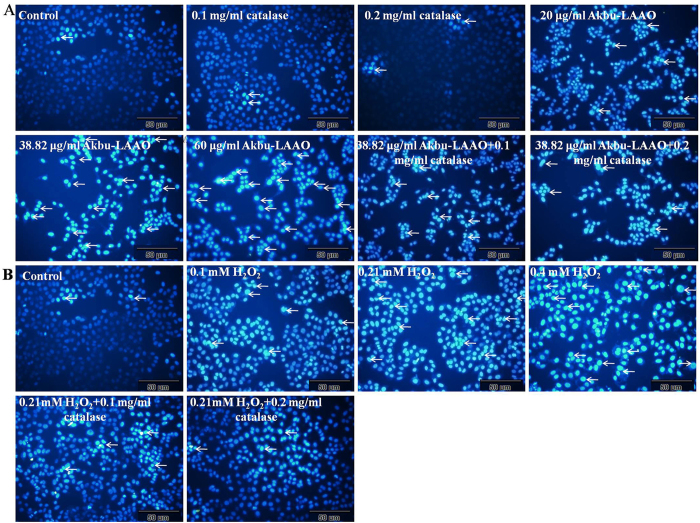
Hoechst 33258 staining assay of HepG2 apoptosis to the stimulations of Akbu-LAAO and exogenous H_2_O_2_. HepG2 cells were incubated in the presence or absence of catalase for 1 h, treated with Akbu-LAAO or exogenous H_2_O_2_ for 24 h and stained Hoechst 33258. Nuclear condensation and/or fragmentation represent cell apoptosis. Images were taken at a magnification of 200×. Arrows marked the apoptotic cells for representing nuclear condensation/fragmentation.

**Figure 6:**
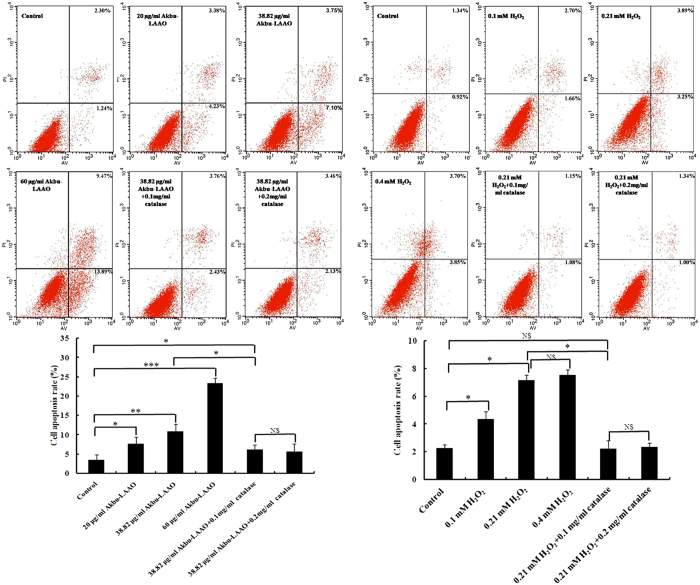
Flow cytometry assay of HepG2 apoptosis induced by Akbu-LAAO and exogenous H_2_O_2_ administrations. The propidium iodide (PI) and FITC-labeled AnnexinV antibody were used for sample labeling reagents. HepG2 cells were incubated in the presence or absence of catalase for 1 h and treated with Akbu-LAAO or exogenous H_2_O_2_ for 24 h. Cells from each group were incubated with Annexin V-FITC and PI, and immediately subjected to flow cytometry assay. Triplicate experiments were performed for each group. *, ** and *** denote *P* < 0.05, 0.01 and 0.001, respectively.

Cell apoptosis of HepG2 cells induced by Akbu-LAAO and H_2_O_2_ was further analyzed by Hoechst 33258 staining and flow cytometry assays. Both assays indicated Akbu-LAAO and exogenous H_2_O_2_ induced apoptosis of HepG2 cells (Figs 5 and 6). Brighter blue staining and more morphological changes were found in nuclear chromatin of HepG2 cells following the treatments of Akbu-LAAO or exogenous H_2_O_2_ for 24 h. Typical morphological characteristics of apoptosis such as nuclear size reduction, cell pyknosis and chromatin condensation were more easily observed in Akbu-LAAO-treated HepG2 than control HepG2 cells (Indicated by arrow, Fig. 5). Hoechst assay showed 0.1 and 0.2 mg/mL of catalase could not induce observable HepG2 apoptosis, which ensures no influence of catalase on the flow cytometry assay. Akbu-LAAO induced the *in vitro* apoptosis of HepG2 cell in a dose-dependent manner. The apoptotic rates of HepG2 cells flowing Akbu-LAAO administration with the dosages of 0, 20, 38.82 and 60 μg/mL for 24 h were measured as ~3.54%, 7.61%, 10.85% and 23.36% (Fig. 6), respectively. The apoptotic rates of HepG2 cells following the treatments of 38.82 μg/mL Akbu-LAAO + 0.1 mg/mL catalase and 38.82 μg/mL Akbu-LAAO + 0.2 mg/mL catalase for 24 h were ~6.19% and 5.59% (Fig. 6). However, the apoptotic rate of HepG2 cells only decreased ~42.95% following the treatment of 38.82 μg/mL Akbu-LAAO + 0.1 mg/mL catalase compared to the HepG2 cells treated with 38.82 μg/mL Akbu-LAAO, which was still 74.86% higher than that of the control HepG2 cells without Akbu-LAAO treatment (Fig. 6). Catalase scavenging only partially reduces Akbu-LAAO-inducible apoptosis of HepG2 cells. In sharp contrast, the apoptotic rates of HepG2 in the presences of 0, 0.1, 0.21, 0.4 mM of H_2_O_2_, 0.21 mM H_2_O_2_ + 0.1 mg/mL catalase and 0.21 mM H_2_O_2_ + 0.2 mg/mL catalase for 24 h were ~2.26%, 4.36%, 7.14%, 7.55%, 2.23% and 2.34%, respectively. Interestingly, no differences were measured for the apoptosis between HepG2 cells without H_2_O_2_ treatment and with 0.21 mM H_2_O_2_ + 0.2 mg/mL catalase treatment (Fig. 6). In another word, catalase scavenging completely restores H_2_O_2_-inducible apoptosis on HepG2 cells. Conclusively, all the above results indicated the cytotoxicity and apoptosis induction of Akbu-LAAO on HepG2 cells are linked to but not solely contributed to the produced H_2_O_2_.

### Akbu-LAAO does not affect the cell cycle of HepG2

**Figure 7:**
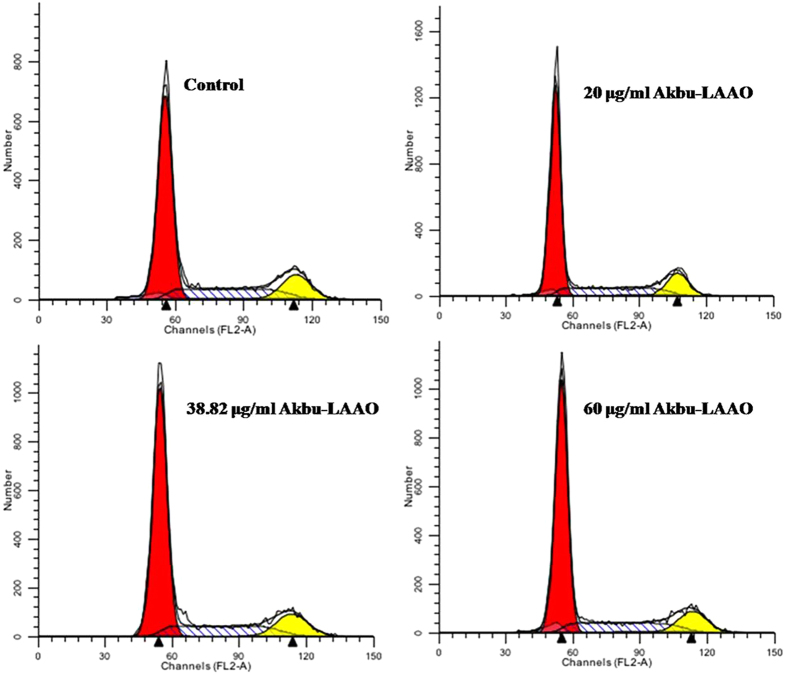
Akbu-LAAO treatment showed no effect on HepG2 cell cycle by flow cytometry assay. Propidium iodide was used as the staining reagent. HepG2 cells were treated with 0, 20, 38.82 and 60 μg/mL of Akbu-LAAO for 24 h at 37 °C with 5% CO_2_. The cells stained with PI were subjected to flow cytometry foe measuring cell phase distributions.

The effect of Akbu-LAAO on HepG2 cell cycle was measured by flow cytometry assay. As shown in Fig. 7, there were no distribution differences of cell populations in G_0_/G_1_, S and G_2_/M phases between HepG2 cells treated with and without Akbu-LAAO. Akbu-LAAO exhibits anti-tumor activity without interrupting HepG2 cell cycle.

### Akbu-LAAO acts on HepG2 cells *via* TGF-β pathway

We screened the differentially expressed genes in HepG2 cells in responding to Akbu-LAAO treatment using Affymetrix Genechip (Human Transcriptome Array 2.0). A total of 254 mRNAs were identified as up- or down-regulated over 1.5- fold in Akbu-LAAO-treated HepG2 cells in comparison with control HepG2 cells. Among these targeting genes, we focused on the molecules involved in TGF-β signal pathway. Gene-gene interaction network analysis indicated the genes *CYR61, p53, GDF15, TOB1, BTG2, BMP2, BMP6, SMAD9, JUN, JUNB, LOX, CCND1, CDK6, GADD45A* and *CDKN1A* in TGF-β signal pathway were apparently differentially expressed following Akbu-LAAO treatment (Table 1), which implicates Akbu-LAAO might exert anti-tumor activity to HepG2 cells *via* TGF-β pathway.

**Figure 8:**
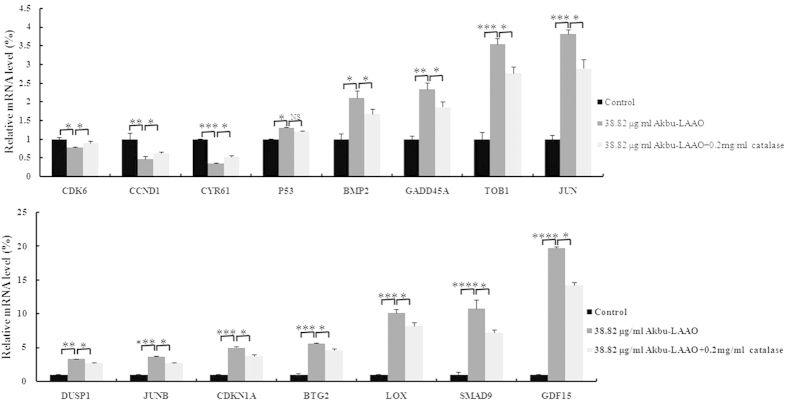
qRT-PCR assay of TGF-β-pathway-related genes deregulated in HepG2 responding to Akbu-LAAO administration. The comparisons of the level changes of targeted genes in HepG2 cells in absence of Akbu-LAAO, in presence of 38.82 μg/mL Akbu-LAAO, in presence of 38.82 μg/ml Akbu-LAAO plus 0.2 mg/ml catalase. *ACTB* was used as the internal reference. The fold changes were represented as mean ± SD from triplicate assays. *, **, *** and **** denote *P* < 0.05, <0.01, <0.001 and <0.0001, respectively.

**Figure 9:**
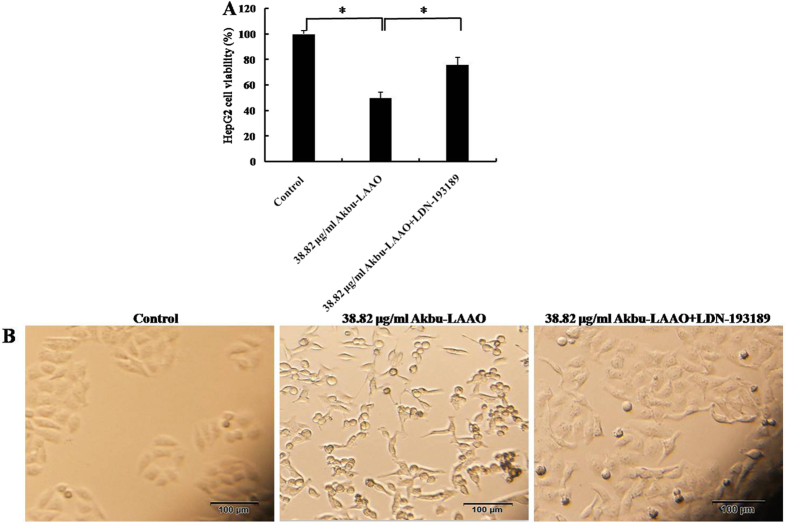
The influence of TGF-β pathway inhibitor LDN-193189 on the cytotoxicity of Akbu-LAAO to HepG2 cells. (**A**) LDN-193189 influence on the cytotoxicity of Akbu-LAAO to HepG2 by MTT assay. (**B**) LDN-193189 influence on the cytotoxicity of Akbu-LAAO to HepG2 cell morphology. HepG2 cells were pre- incubated with 10 μM LDN-193189 for 1 h and treated with 38.82 μg/mL Akbu-LAAO for 24 h. Experiments were performed in triplicate, * denotes *P* < 0.05

The expression level changes of above 15 genes in HepG2 cells in responding to Akbu-LAAO were further validated by qRT-PCR analysis. In consistent with the microarray results, qRT-PCR data revealed the same trend for the level changes of these genes in HepG2 cells following Akbu-LAAO treatment (Fig. 8, Table 1). We also checked the effect of catalase on the expression levels of these genes. 0.2 mg/mL catalase could only slightly restore the level changes of mRNAs in HepG2 induced by Akbu-LAAO (Fig. 8, Table 1), which again implicated the presence of other action factor for endowing Akbu-LAAO the cytotoxicity to HepG2 except for H_2_O_2_ production. It is necessary to confirm Akbu-LAAO might exert anti-tumor activity to HepG2 through TGF-β pathway. The treatment of LDN-193189, a TGF-β pathway inhibitor, could decrease the cytotoxicity of Akbu -LAAO (38.82 μg/mL) to HepG2 cells by ~52% in 24 h by MTT assay (Fig. 9A). In addition, morphological changes of HepG2 induced by Akbu-LAAO could be restored to certain extent in the presence of LDN-193189 (Fig. 9B). Taken together, current work concluded Akbu-LAAO exhibits potent anti-tumor activity to HepG2 cells partially through produced H_2_O_2_ and *via* TGF-β signal pathway.

## Discussion

LAAOs play important roles in the biological activities for snake venom (SV). SV-LAAOs induce platelet aggregation and cell apoptosis, exhibit anti-microbial, anti-leishmaniasis, anti-tumor and anti-HIV activities^7^,^8^,^9^,^10^,^11^,^12^,^13^,^14^,^15^. SV-LAAOs were reported to exhibit cytotoxicity and apoptosis-induction towards leukemia HL-60^17^,^19^ and K562 cells^20^, gastric carcinoma AGS and breast carcinoma MCF-7 cells^21^, colorectal carcinoma RKO cells^14^, melanoma B16 cells^17^, cervical carcinoma Hela cells^20^ and lung carcinoma A549 cells^22^.

Previously, we purified an LAAO (Akbu-LAAO) from *Agkistrodon blomhoffii ussurensis* snake venom and characterized its biological properties^16^. Current work demonstrated that Akbu-LAAO inhibited the *in vitro* proliferation (Fig. 1) and induced the apoptosis of HepG2 cells (Figs 5 and 6). It showed cytotoxicity toward HepG2 cells with an IC_50_ of 38.82 μg/mL (~0.3 μM, Fig. 1) that was ~700 folds lower than that of exogenous H_2_O_2_ (~0.21 mM, Fig. 2C). H_2_O_2_ is commonly believed to play an important role in the anti-tumor activities of SV-LAAOs. LAAOs could bind directly to cell surface. The enzymatic-released H_2_O_2_ accumulates at the localized area to a relative higher concentration to trigger cell apoptosis^18^,^23^. The anti-tumor activities of SV-LAAO were reported to be inhibited by catalase and other H_2_O_2_ scavengers^8^,^9^,^18^. Current work indicated that the anti-tumor activity of Akbu-LAAO toward HepG2 differed from exogenous H_2_O_2_. It inhibited the *in vitro* proliferation of HepG2 partially through enzymatic-released H_2_O_2_ (Fig. 2B) compared with exogenous H_2_O_2_ (Fig. 2D). So, except for H_2_O_2_ action mechanism, Akbu-LAAO might act on HepG2 through unknown path.

We also found the difference of HepG2 apoptosis induced by Akbu-LAAO and exogenous H_2_O_2_ by Hoechst staining and flow cytometry assays (Figs 5 and 6). Comparing to the Akbu-LAAO-treated HepG2 cells (Fig. 5A), the addition of catalase significantly inhibited the apoptosis of HepG2 cells induced by exogenous H_2_O_2_ (Fig. 5B). 0.1 mg/mL catalase decreased the apoptotic rate of HepG2 cells treated with 38.82 μg/mL Akbu-LAAO by ~42.95% that was 74.86% higher than the cells receiving no Akbu-LAAO treatment (Fig. 6). In the sharp contrast, in the presence of 0.1 mg/mL catalase, the apoptosis rate of HepG2 receiving 0.21 mM H_2_O_2_ stimulation was 2.23% comparable to 2.26% of HepG2 cells without H_2_O_2_ stimulation (Fig. 6). The cytotoxicity of Akbu-LAAO to HepG2 cells is partially linked to enzymatic-released H_2_O_2_.

Akbu-LAAO shows no effect on the cell cycle of HepG2. *B. atrox* LAAO was reported to arrest HL-60 cells at G_0_/G_1_ phase by delaying its progression to S and G2/M phases^19^. *A. acutus* LAAO (ACTX-6) could markedly increase cell accumulation at sub-G1 phase^24^. Interestingly, our results showed Akbu-LAAO did not affect the cell cycle of HepG2 cells (Fig. 7). Akbu-LAAO exhibits anti-tumor activity on HepG2 cells by inhibiting cell proliferation and inducing cell apoptosis without disturbing cell cycle.

**Figure 10:**
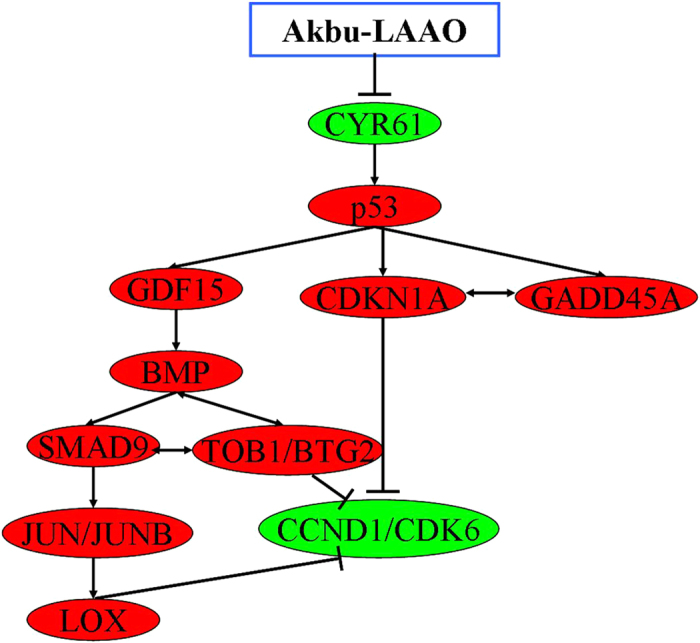
Potential action mechanism of Akbu-LAAO on HepG2 proliferation and apoptosis. Red nodes represent upregulated genes, green nodes represent downregulated genes, 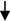 represents activation, ⊥ represents inhibition, ↔ represents interaction between genes.

To reveal the cytotoxic action mechanism of Akbu-LAAO to HepG2 cells, the differentially expressed genes in HepG2 cells in responding to Akbu-LAAO stimulation were screened using cDNA microarray. Comparing with control HepG2 cells, 254 mRNAs were found up- or down- regulated over 1.5-fold in Akbu-LAAO treated HepG2 cells (Data unshown). 15 genes including *CYR61, p53*, *GDF15, TOB1, BTG2, BMP2, BMP6, SMAD9, JUN, JUNB, LOX, CCND1, CDK6, GADD45A* and *CDKN1A* involved in TGF-β signaling pathway were selected as potential targeting genes in HepG2 cells toward Akbu-LAAO (Table 1). A potential action mechanism of Akbu-LAAO to HepG2 *via* TGF-β pathway was proposed by the genetic and qRT-PCR results as schemed in Fig. 10. 0.2 mg/mL catalase only slightly restored the level changes of 15 genes induced by Akbu-LAAO treatment in HepG2 (Fig. 8, Table 1), implicating H_2_O_2_ production was partially responsible for Akbu-LAAO’s cytotoxicity to HepG2 cells. In addition, LDN-193189, a TGF-β pathway inhibitor, decreased the cytotoxicity of 38.82 μg/mL Akbu-LAAO to HepG2 cells by ~52% (Fig. 9A). LDN-193189 treatment could restore the morphological change of HepG2 induced by Akbu-LAAO to certain extent (Fig. 9B). The above results suggest Akbu-LAAO exhibiting potent anti-tumor activity to HepG2 cells partially through produced H_2_O_2_ *via* TGF-β pathway.

TGF-β pathway was associated with tumor cell proliferation, differentiation, apoptosis, tumor occurrence and development^25^. CYR61, p53, TOB1/BTG2 and CCND1/CDK6 are critical molecules in TGF-β signaling pathway. Based on the validated targeting genes differentially expressed in HepG2 cells following Akbu-LAAO treatment and summarized results from published literatures, we propose Akbu-LAAO acts on HepG2 mainly *via* the following detailed pathways.

1. CYR61-p53-CDKN1A-CCND1/CDK6 pathway: As a tissue growth factor, the activation of CYR61 promoted tumor cell proliferation and inhibited apoptosis by inhibiting p53 expression^26^,^27^. In case of cell damage, the increased expression of p53 induced CDKN1A upregulation^28^. CCCND1 could form complex with CDK4 and CDK6 whose overexpression promoted cell cycle progression and cancer development^29^,^30^. The tumor suppression activity of CCCND1/CDK6 was negatively correlated with CDKN1A level^29^,^30^. Current work showed Akbu-LAAO treatment decreased *CYR61* level, consequently might enhance mRNA levels of p53 and *CDKN1A*, finally suppressed the level of *CCCND1/CDK6* (Table 1 and Fig. 10) to inhibit HepG2 proliferation and induce HepG2 apoptosis, which suggests its action to HepG2 cells *via* CYR61-p53-CDKN1A-CCND1/CDK6 mechanism.
2. CYR61-p53-GADD45A-CDKN1A-CCND1/CDK6 pathway: As a downstream target for p53, GADD45A acts as a tumor suppressor by keeping genomic stability through the interactions with CDKN1A and PCNA^31^,^32^. GADD45A overexpression can induce tumor cell apoptosis and decrease tumor cell proliferation, survival and tumorigenesis^33^. Our work showed *GADD45A* (Table 1) was significantly up-regulated in HepG2 cells following Akbu-LAAO treatment. The activation of *p53* on *GADD45A* together with the intermediation of *GADD45A* and *CDKN1A* potentially inhibit the expression of *CCND1/CDK6* (Table 1), which suppresses the proliferation and induces the apoptosis of HepG2. It suggested Akbu-LAAO potentially mediates HepG2 proliferation and apoptosis *via* CYR61-p53-GADD45A-CDKN1A-CCND1/CDK6, as schemed in Fig. 10.
3. CYR61-p53-GDF15-BMP-SMAD9-JUN/JUNB-LOX-CCND1/CDK6 path: GDF and BMP are members of the TGF-β superfamily^34^,^35^. The activation of GDF-15 and BMP by p53 could enhance the expression of SMAD by receiving TGF-β-induced signals from cell surface to nucles^35^ to regulate JUN/JUNB transcription^36^,^37^. As an extracellular matrix (ECM)^38^,^39^ remodeling enzyme, enhanced LOX activity by SMAD and JUNB^40^ downregulated CCND1/CDK6 expression to inhibit cell proliferation and induce cell apoptosis^41^. Current work showed the mRNA levels of *GDF-15, BMP2, SMAD9, JUN/JUNB* and *LOX* were significantly elevated in HepG2 following Akbu-LAAO treatment (Table 1). Akbu-LAAO potentially suppresses cell proliferation and induces apoptosis *via* CYR61-p53-GDF15-BMP-SMAD9-JUN/JUNB-LOX-CCND1/CDK6 path (Fig. 10).
4. CYR61-p53-GDF15-BMP-(SMAD9)-TOB1/BTG2-CCND1/CDK6 pathway: TOB/BTG family is involved in cell growth, differentiation and survival by adjusting and balancing BMP signaling^42^,^43^. BMP2 induces TOB/BTG transcription. Interestingly, it was reported that TOB negatively and BTG2 positively correlated with SMAD in BMP signal transduction^44^, respectively. BMP signaling mediates the relative levels of SMAD inhibitors and activators in cells^45^. BTG/TOB inhibited cell proliferation and induced cell apoptosis *via* reducing CCND1/CDK6^45^. In this work, *BTG2* and *TOB1* were up-regulated and *CCND1/CDK6* were down-regulated in HepG2 cells following Akbu-LAAO treatment, which suggests Akbu-LAAO also exhibiting cytotoxicity to HepG2 cells *via* CYR61-p53-GDF15-BMP-(SMAD9)-TOB1/BTG2-CCND1/CDK6 path (Fig. 10).

## Conclusions

Akbu-LAAO significantly inhibits the *in vitro* proliferation and induces the apoptosis of HepG2 cells without interrupting its cell cycle. Akbu-LAAO administration can induce morphology and ultrastructure changes of HepG2 cells. The cytotoxicity of Akbu-LAAO towards HepG2 cells is partially linked to enzymatic-produced H_2_O_2_. Gene microarray, qRT-PCR and TGF-β pathway activity blocking assays prove that Akbu-LAAO exerts tumor suppression effect on HepG2 cells *via* TGF-β pathway. The current study suggests Akbu-LAAO as a potential anti-tumor drug and provides new clues to anti-tumor action mechanism for SV-LAAOs.

## Methods

### Materials

RPMI 1640 and pancreatin were from Gibco (USA). Fetal bovine serum (FBS) was from TransGen (China). Trizol^TM^ reagent was from Life (USA). PrimeScript^TM^ RT reagent kit with gDNA eraser was from TaKaRa (Japan). FastStart universal SYBR green master and BrdU assay ELISA kit were from Roche (Switzerland). Annexin V-FITC/propidium iodide apoptosis detection kit, Hoechst 33258 and cell cycle detection kit were from KeyGEN (China). Agilent M × 3005P real-time PCR machine was from Agilent (USA). LDN-193189 was from Selleckchem (USA). FACSCalibur flow cytometry was from BD Biosciences (USA). Olympus IX71-A12FL/PH fluorescence microscope and JEOL JEM-1200EX transmission electron microscopy were from Japan. Microplate reader and Nanodrop 2000 were from Thermo Scientific (USA).

### Cell culture

Human hepatocellular carcinoma HepG2 cells were cultured in 90% RPMI 1640 supplemented with 10% FBS, 100 U/mL penicillin and 100 U/mL streptomycin in a humidified environment at 37 °C with 5% CO_2_.

### Cell viability determination by MTT assay

The influence of Akbu-LAAO or exogenous H_2_O_2_ on the viability of HepG2 cells was determined by MTT assay. HepG2 cells were seeded into a 96-well plate at the density of 7 × 10^3^ cells/well in 100 μL medium and incubated at 37 °C with 5% CO_2_ overnight. The cells were then treated with different concentrations of Akbu-LAAO (0, 5, 10, 20, 40, 80 μg/mL), exogenous H_2_O_2_ (0, 0.05, 0.1, 0.2, 0.4, 0.8, 1.6, 3.2 mM) or catalase (0.1, 0.2, 0.4, 0.6, 0.8 mg/mL), at 37 °C, 5% CO_2_ for 12, 24 or 48 h. Then the medium was replaced with 5 mg/mL MTT solution and incubated for 4 h in darkness at 37 °C, 5% CO_2_. 150 μL of DMSO was added into each well and the absorbances at 570 nm was measured using a microplate reader (Thermo, USA). Results are the averages from triplicate measurements.

The influences of catalase or LDN-193189 (TGF-β signaling pathway inhibitor) on the cytotoxicities of Akbu-LAAO or exogenous H_2_O_2_ to HepG2 was measured using MTT assay. HepG2 cells were pre-incubated with 0, 0.1, 0.2 mg/mL catalase or 10 μM LDN-193189 and treated with 38.82 μg/mL Akbu-LAAO or 0.21 mM H_2_O_2_ for 24 h at 37 °C with 5% CO_2_. The rest steps were the same as described above.

### Cell proliferation by BrdU assay

The influence of Akbu-LAAO or exogenous H_2_O_2_ on the proliferation of HepG2 was determined by BrdU assay. Being treated with 0, 5, 10, 20, 40, 80 μg/mL of Akbu-LAAO for 24 h at 37 °C with 5% CO_2_, HepG2 cells were incubated with 20 μL BrdU labeling solution per well for 4 h, fixed and incubated with anti-BrdU mAb according to the manufacturer’s instruction. Finally, the absorbance at 450 nm was measured using a microplate reader. Results were the averages from triplicate measurements.

### Cell morphology assay

The effect of Akbu-LAAO or exogenous H_2_O_2_ on HepG2 morphology was measured by an inverted light microscope. 7 × 10^3^ cells/well were seeded into a 96-well plate and incubated at 37 °C, 5% CO_2_ overnight. Then, the cells were administrated with 20, 38.82, 60 μg/mL Akbu-LAAO or 0.1, 0.21, 0.4 mM exogenous H_2_O_2_ in the presence or absence of catalase for 24 h. Cell morphology images were taken at the magnification of 100×.

### Cell ultrastructure assay

Transmission electron microscopy (TEM) was used to characterize the ultrastructure alteration of HepG2 cells caused by Akbu-LAAO stimulation. Being incubated in 20, 38.82, 60 μg/mL Akbu-LAAO for 24 h, the HepG2 cells were collected, washed with pre-cooled PBS, and fixed in 2.5% (v/v) glutaraldehyde at 4 °C for 24 h. The slices were stained with 2% osmic acid for 2 h at RT, washed with PBS twice, dehydrated in a graded series of ethanol 50%, 70%, 80%, 90%, 100%, 100%, substituted with propylene and embedded in epoxy resin for 6 h. Ultrathin sections (60–90 nm) were observed and photographed on a JEOL JEM-1200EX system (Japan) operated at 100 KV. Images were digitally acquired from 5 fields randomly selected for each condition.

### Hoechst 33258 staining assay

Hoechst 33258 staining was performed to capture apoptotic induction of Akbu-LAAO or exogenous H_2_O_2_ to HepG2 cells. Cells were seeded into a 96-well plate at a density of 7 × 10^3^ cells/well in 100 μL medium and incubated at 37 °C with 5% CO_2_ overnight. The cells were then treated with 20, 38.82, 60 μg/mL Akbu-LAAO or 0.1, 0.21, 0.4 mM exogenous H_2_O_2_ in the presence or absence of catalase for 24 h, washed with PBS for 3 times and fixed with 4% paraformaldehyde for 30 min at 4 °C. Being washed with PBS for 3 times, HepG2 cells were stained with Hoechst 33258 solution in the dark for 10 min at RT. The cells were washed with PBS for 3 times and immediately imaged by an inverted fluorescence microscope with the excitation wavelength of 340 nm at 200×.

### Flow cytometry assay

Flow cytometry assay was performed to investigate the apoptosis induction of Akbu-LAAO to HepG2 cells. 1 × 10^6^ cells were seeded into a 6-cm dish and incubated at 37 °C with 5% CO_2_ overnight. Following the treatments of 20, 38.82, 60 μg/mL Akbu- LAAO or 0.1, 0.21, 0.4 mM exogenous H_2_O_2_ with or without the presence of catalase at 37 °C, 5% CO_2_ for 24 h, the corresponding HepG2 cells from each group were harvested with trypsin digestion, washed with PBS for 3 times and centrifuged at 1000 rpm for 5 min. The obtained cell pellets were resuspended in 500 μL binding buffer, incubated in 5 μL Annexin V-FITC (FITC-labeled Annexin V antibody) and 5 μL PI in the dark for 30 min at RT, immediately subjected to flow cytometry and analyzed with Cell Quest software.

### Cell cycle assay

Propidium iodide (PI) staining assay was used to analyze the influence of Akbu-LAAO on HepG2 cycle. 1 × 10^6^ HepG2 cells were seeded into a 6-cm dish and incubated at 37 °C with 5% CO_2_ overnight. The cells were then administrated with 20, 38.82, 60 μg/mL of Akbu-LAAO at 37 °C, 5% CO_2_ for 24 h, washed twice with PBS buffer and digested with trypsin (EDTA free). Cells were collected by centrifuging at 1000 rpm for 5 min and washed with PBS. The cell pellets were resuspended and fixed with ice-cold 70% ethanol at 4 °C overnight. Cell pellets were obtained by aspirating ethanol, washed with PBS, incubated with 100 μL RNase A at 37 °C for 30 min and labeled in 400 μL PI at 4 °C for 30 min in the dark. Cell cycle was analyzed using flow cytometry and analyzed using CellQuest software.

### cDNA microarray screening targeting genes of Akbu-LAAO in HepG2 cells

1 × 10^7^ HepG2 and 1 × 10^7^ Akbu-LAAO-treated (38.82 μg/mL Akbu-LAAO at 37 °C, 5% CO_2_ for 24 h) HepG2 cells were harvested for total RNA extraction using Trizol^TM^ reagent (Life Sciences). The RNA concentration and quality were assessed by NanoDrop 2000 spectrophotometer (Thermo) and 1.5% denaturing agarose gel electrophoresis. cDNA was synthesized using SuperScript II kit and purified by QIAGEN RNeasy Mini Kit. cRNA was created using a Genechip IVT Labeling Kit. The biotin- labeled fragmented cRNA (≤200 nt) was hybridized at 45 °C for 16 h to Affymetrix Genechip (Human Transcriptome Array 2.0). All the arrays were washed, imaged by 3000 7G Scanner and proceeded by Affymetrix Genechip Operating Software. Random variance model (RVM) t-test was performed to screen the differentially expressed genes from independent triplicate experiments.

### Quantitative real-time PCR (qRT-PCR)

qRT-PCR was performed to determine the level changes of targeted genes following Akbu-LAAO treatment. Total RNA was extracted from HepG2 cells using Trizol ^TM^ reagent. Reverse transcription was performed using PrimeScript^TM^ RT Kit with gDNA Eraser. PCR was carried out on an Agilent M × 3005P real-time PCR machine. β-actin (*ACTB*) was used as the internal reference. PCR primers for targeted genes were listed in Table 2. The comparison of mRNA expression level was calculated using 2^−ΔΔCT^ method^46^.

### Data processing and statistical analysis

SPSS 17.0 software was utilized for data analysis. Results are represented as mean ± SD of at least three independent experiments. The differences were assessed with student’s *t* test. Values with *P < 0.05, **P < 0.01, ***P < 0.001 and ****P < 0.0001 were considered statistically significant differences.

## Additional Information

**How to cite this article**: Guo, C. *et al.* Akbu-LAAO exhibits potent anti-tumor activity to HepG2 cells partially through produced H_2_O_2_ via TGF-β signal pathway. *Sci. Rep.* **5**, 18215; doi: 10.1038/srep18215 (2015).

## Figures and Tables

**Table 1:** Generic microarray and qRT-PCR determinations of the mRNA expression levels of deregulated genes involved in TGF-β signal pathway in responding to Akbu-LAAO.

Gene symbol	fold ratio (Akbu-LAAO-microarray)^a^	fold ratio (Akbu-LAAO-qRT-PCR)^b^	fold ratio (Akbu-LAAO + catalase-qRT-PCR)^c^
*CDK6*	0.59	0.77	0.89
*CCND1*	0.62	0.48	0.63
*CYR61*	0.53	0.36	0.52
*P53*	1.58	1.30	1.20
*BMP2*	1.76	2.10	1.67
*GADD45A*	1.87	2.33	1.56
*TOB1*	1.61	3.54	2.46
*JUN*	1.54	3.82	2.79
*DUSP1*	1.54	3.29	2.65
*JUNB*	1.60	3.63	2.67
*CDKN1A*	1.83	4.99	3.78
*BTG2*	1.78	5.59	4.65
*LOX*	3.18	10.09	7.23
*SMAD9*	1.66	10.73	6.25
*GDF15*	2.33	19.75	14.23

^a,b^Refer to mRNA level changes of deregulated genes in HepG2 cells in responding to Akbu-LAAO treatment compared with control HepG2 cells; ^c^Refers to the relative mRNA levels of deregulated genes in HepG2 cells in responding to Akbu-LAAO treatment plus catalase compared with control HepG2 cells.

**Table 2:** Primers designed for deregulated genes in HepG2 following Akbu-LAAO treatment.

Gene symbol	Primer sequence	Product size (bp)
*ACTB*	F:5′-AGGCCAACCGCGAGAAG-3′	181
	R:5′-ACAGCCTGGATAGCAACGTACA-3′	
*CCND1*	F:5′-GTACCCCGATGCCAACCTCC-3′	238
	R:5′-TTTCACGGGCTCCAGCGACA-3′	
*CDK6*	F:5′-ACTTTCTTCATTCACACCGAGT-3′	83
	R:5′-GAGTTTTATTTGTCCGCTGCT-3′	
*CYR61*	F:5′-AGCCTCGCATCCTATACAACC-3′	143
	R:5′-TTCTTTCACAAGGCGGCACTC-3′	
*GDF15*	F:5′-CCGCCAGCTACAATCCCAT-3′	81
	R:5′-TGGCTAACAAGTCATCATAGGTC-3′	
*JUN*	F:5′-GCTGCCTCCAAGTGCCGAAA-3′	133
	R:5′-TAAGCTGTGCCACCTGTTCCC-3′	
*LOX*	F:5′-GATTTCTTACCCAGCCGACCA-3′	230
	R:5′-TAACAGCCAGGACTCAATCCC-3′	
*SMAD9*	F:5′-AGTCAGTTCACCACGGCTTT-3′	92
	R:5′-ATACTCAGCACCCCAACCCT-3′	
*TOB1*	F:5′-AAAAGCCATACAAAGGATCGG-3′	210
	R:5′-TATCATCCACGTAAAGCACCT-3′	
*p53*	F:5′-TCCTCAGCATCTTATCCGAGT-3′	233
	R:5′-TCCGTCCCAGTAGATTACCAC-3′	
*DUSP1*	F:5′-CATCAGCTCCTGGTTCAACGA-3′	180
	R:5′-CGCCTCTGCTTCACAAACTCA-3′	
*CDKN1A*	F:5′-CACTGTCTTGTACCCTTGTGC-3′	89
	R:5′-CCGCCGTTTTCGACCCTGA-3′	
*GADD45A*	F:5′-CAGAAGACCGAAAGCGACCC-3′	130
	R:5′-TGATGTCGTTCTCGCAGCAA-3′	
*BTG2*	F:5′-CCCCTATGAGGTGTCCTACCG-3′	159
	R:5′-CTGGAGACTGCCATCACGTA-3′	
*JUNB*	F:5′-ATGGAACAGCCCTTCTACCAC-3′	208
	R:5′-AGCCCTGACCAGAAAAGTAGC-3′	
*BMP2*	F:5′-CAATAGCAGTTTCCATCACCGAA-3′	203
	R:5′-CCACTTCCACCACGAATCCAT-3′	
